# Advances in immunotherapy for uveal melanoma: enhancing efficacy and overcoming resistance

**DOI:** 10.3389/fcell.2025.1619150

**Published:** 2025-06-30

**Authors:** Jian Song, Pei Mou, Guo-Ge Song, Liang Chen, Yu-Qing Chen, Rui-Li Wei

**Affiliations:** ^1^ Department of Ophthalmology, Changzheng Hospital of Naval Medical University, Shanghai, China; ^2^ Department of Ophthalmology, No. 906 Hospital of People’s Liberation Army, Ningbo, China; ^3^ Department of Anesthesiology, Changzheng Hospital of Naval Medical University, Shanghai, China; ^4^ Department of Ophthalmology, People’s Liberation Army Navy 971 Hospital, Qingdao, China

**Keywords:** uveal melanoma, immunotherapy resistance, tumor microenvironment, predictive biomarkers, immune checkpoint inhibitors

## Abstract

Uveal melanoma (UM) is the most common primary intraocular malignancy in adults, presenting a significant clinical challenge due to its high metastatic potential and limited response to conventional systemic therapies. While immunotherapy has transformed the treatment landscape for numerous cancers, its effectiveness in UM has been substantially limited, primarily due to the tumor’s distinct immune-evasive characteristics and a suppressive tumor microenvironment (TME). This review systematically examines the multiple mechanisms underlying immunotherapy resistance in UM, including low tumor mutational burden, immune checkpoint overexpression, metabolic adaptations, and the epigenetic silencing of immune-stimulatory genes. Additionally, we analyze emerging strategies aimed at modifying the TME to enhance immune recognition and response, which include targeting suppressive immune cell populations, addressing metabolic and hypoxic barriers, and utilizing epigenetic modulators to restore immune activation pathways. Furthermore, we highlight recent advances in identifying predictive biomarkers—such as genetic mutations (e.g., *BAP1*, *MBD4*), immune gene signatures, circulating tumor DNA, and protein-based blood markers—that may facilitate patient stratification and treatment selection. We also examine novel combination approaches that integrate immune checkpoint inhibitors with targeted therapies, radiation, metabolic interventions, or engineered cellular therapies, several of which have shown promising clinical potential in overcoming UM’s inherent resistance mechanisms. Despite persistent challenges, such as toxicity management and limited availability of large-scale trials due to UM’s rarity, the integration of multi-omics profiling, precision medicine frameworks, and adaptive trial designs presents new opportunities for therapeutic advancement. This review provides a translational perspective on enhancing immunotherapy efficacy in UM by addressing its unique biology and identifying future directions for clinical innovation.

## 1 Introduction

Uveal melanoma (UM), the most prevalent primary intraocular malignancy in adults, presents significant therapeutic challenges due to its metastatic propensity, particularly to the liver, and poor prognosis in advanced stages (Jager et al., 2017). While localized disease can often be managed effectively with radiation or surgical intervention, treatments for metastatic UM have historically demonstrated limited efficacy, with median survival typically <1 year (Bol et al., 2019). The advent of immunotherapy, particularly immune checkpoint inhibitors (ICIs), has revolutionized cancer treatment by harnessing the immune system’s antitumor capabilities. However, UM exhibits marked resistance to these approaches, with response rates remaining <15% in clinical trials—a stark contrast to the significantly higher efficacy observed in cutaneous melanoma (Heppt et al., 2017b; Komatsubara and Carvajal, 2017). This resistance underscores the critical need to explore UM’s distinct immunological features and develop strategies to overcome both intrinsic and acquired resistance mechanisms.

A key barrier to effective treatment lies in UM’s tumor microenvironment (TME). Unlike cutaneous melanoma, UM is characterized by an immunologically “cold” TME, marked by a low tumor mutational burden, limited T-cell infiltration, and an abundance of immunosuppressive cell populations (Kaler et al., 2022; Wang et al., 2020). Genetic analyses have implicated specific mutations—present in approximately half of UM cases—in the upregulation of immunosuppressive factors such as *PROS1*, which inhibits dendritic cell maturation and promote immune tolerance (Figueiredo et al., 2020; Kaler et al., 2022). Moreover, UM cells evade immune detection through increased expression of immune checkpoint molecules like PD-L1, further dampening antitumor immune responses (Lamas et al., 2023; Stålhammar et al., 2019). These insights demonstrate how genetic alterations and immune-evasion strategies cooperate to maintain UM’s resistance to conventional immunotherapies. The complex interplay between microenvironmental and molecular mechanisms creates multiple layers of resistance to current therapeutic approaches.

Recent investigations underscores the importance of predictive biomarkers for identifying patients who may benefit from immunotherapy. For instance, specific immune-related genetic signatures, including those involving apoptosis-related genes or distinct lymphocyte populations, have demonstrated potential prognostic value (Cao et al., 2021; Sun et al., 2021). Similarly, serum protein signatures and circulating tumor DNA (ctDNA) are being evaluated as minimally invasive methods for monitoring treatment response (Herrspiegel et al., 2023; Francis et al., 2024). However, clinical implementation remains limited due to methodological inconsistencies and a lack of standardized validation.

Novel therapeutic combinations are increasingly recognized as strategies to overcome treatment resistance. Preclinical and clinical studies suggest that combining ICIs with radiotherapy, targeted agents, or metabolic modulators can improve treatment efficacy. Notably, an experimental melanocyte-targeted therapy demonstrated significant survival benefits in selected metastatic patients with UM, with survival rate of approximately 75% beyond 1 year (Damato et al., 2019; Dimitriou et al., 2025). Additional approaches combining immunotherapy with liver-directed therapies or epigenetic modifiers aim to modify the host environment and enhance immune cell function (Jespersen et al., 2019; Montazeri et al., 2023). Furthermore, although still in early stages, cell-based therapies engineered to target UM-specific antigens have shown preliminary efficacy in preclinical models (Synoradzki et al., 2024).

Despite these advances, substantial challenges remain. UM’s rarity and unique biological characteristics complicate the direct translation of therapeutic strategies from other cancer types. Additionally, determining optimal treatment sequencing and managing immune-related adverse events remain critical considerations requiring further investigation (Koch et al., 2022; Khimani et al., 2022). This review synthesizes recent advances in understanding immunotherapy resistance in UM, explores novel approaches to TME modulation, evaluates predictive biomarkers, and examines emerging combination strategies. Through this analysis, we aim to outline practical approaches for improving response rates and survival outcomes in this challenging disease.

## 2 Intrinsic resistance mechanisms in UM

UM exhibits both intrinsic and acquired resistance to immunotherapy treatments, driven by multiple interconnected factors, including genetic alterations, environmental interactions, and characteristics of the cellular microenvironment. Understanding these mechanisms is crucial for developing strategies to overcome treatment resistance.

### 2.1 Suppressive tumor environment characteristics

The cellular environment in UM demonstrates limited immune activity, characterized by low infiltration of effector immune cells and elevated levels of immunosuppressive cell populations. Myeloid-derived suppressor cells and regulatory T-cells dominate in these cases, secreting inhibitory cytokines such as interleukin-10 (IL-10) and transforming growth factor-beta (TGF-β), which suppress effector immune cell function and promote immune tolerance (Kaler et al., 2022; Wang et al., 2020). Genetic analyses reveal that *BAP1* mutations—present in approximately half of patients with UM—lead to increased expression of the PROS1 protein, which inhibits dendritic cell development and contributes to an immunosuppressive microenvironment (Figueiredo et al., 2020; Kaler et al., 2022). Furthermore, UM cells secrete extracellular vesicles containing microRNAs (such as miR-146a), which further compromise immune surveillance mechanisms (Dong et al., 2022).

### 2.2 Limited genetic variation and target deficiency

UM exhibits an exceptionally low mutational burden compared with other solid tumors, with approximately 0.5 mutations per DNA segment. This low mutation rate reduces the likelihood of producing immunogenic neoantigens (Komatsubara and Carvajal, 2017). Primary mutations in *GNAQ/GNA11* lack immunogenicity, and the absence of UV-induced DNA damage—prevalent in other cancers—further reduces antigenic diversity (Larribère and Utikal, 2020). This molecular profile renders UM effectively “invisible” to immune surveillance mechanisms. Consequently, ICI therapies targeting PD-1/CTLA-4 pathways, which typically require preexisting immune recognition of cancer cells, often yield poor responses (Heppt et al., 2017a).

**FIGURE 1:**
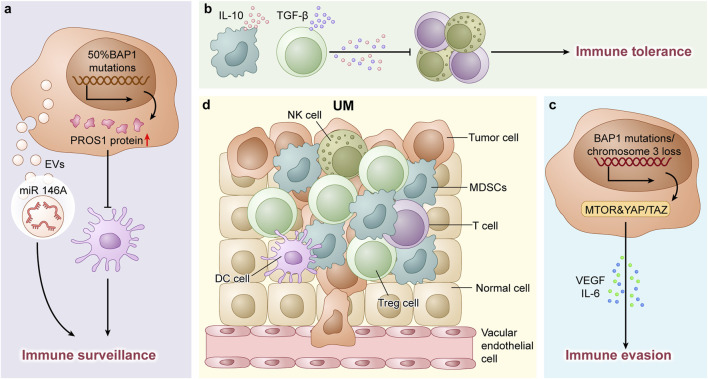
**(a)** In UM, 50% of cases harbor *BAP1* gene mutations, which lead to upregulated PROS1 protein expression. This subsequently suppresses dendritic cell differentiation and contributes to the formation of an immunosuppressive microenvironment. Additionally, UM-derived extracellular vesicles (EVs) carrying microRNAs (e.g., miR-146a) further disrupt immune surveillance mechanisms. **(b)** The tumor immune microenvironment in UM is predominantly characterized by myeloid-derived suppressor cells (MDSCs) and regulatory T-cells (Tregs). These immunosuppressive populations secrete inhibitory cytokines such as IL-10 and TGF-β, which impair effector immune cell functions and promote immune tolerance. **(c)** In UM, tumors with intact *BAP1* or monosomy 3 exhibit activation of oncogenic pathways (e.g., mTOR and YAP/TAZ), which drive the production of immunosuppressive Factors (such as VEGF and IL-6), thereby promoting tumor progression and immune evasion. **(d)** Schematic Illustration of Cellular Components in the TME of UM.

Regarding genetic factors, in certain patients with UM, hypermutated tumors result in DNA repair deficiencies. Paradoxically, these tumors may demonstrate enhanced responsiveness to immune checkpoint therapies, potentially due to increased neoantigen presentation (Saint-Ghislain et al., 2022). Conversely, tumors with intact *BAP1* or chromosome 3 loss display chromosomal instability and activate oncogenic pathways such as mTOR and YAP/TAZ. These pathways induce the production of immunosuppressive factors such as VEGF and IL-6, supporting tumor progression and immune evasion (Durante et al., 2020; Kaler et al., 2022). Epigenetic regulators such as EZH2 are also upregulated in UM, suppressing tumor-suppressor genes and inhibiting T-cell infiltration—a phenomenon recently identified as potentially targetable by specific therapies (Li et al., 2025). The immunosuppressive microenvironment characteristics of UM are illustrated in the Figure 1.

### 2.3 Overactive immune checkpoints and alternative inhibition

UM demonstrates elevated expression of immune checkpoint molecules and activation of alternative inhibitory pathways. Specifically, increased expression of LAG-3 and TIM-3 checkpoint proteins adds additional layers of immune suppression beyond the PD-1/CTLA-4 axis (Lamas et al., 2023; Stålhammar et al., 2019). Tumor cells may also activate alternative signaling pathways involving molecules such as indoleamine 2,3-dioxygenase (IDO1) or adenosine, altering the local metabolic environment to inhibit immune cell function. These overlapping pathways create redundancy in immune-evasion strategies, necessitating combinatorial approaches that will be discussed in subsequent sections.

UM cells evade immune detection by overexpressing checkpoint molecules beyond PD-L1, including LAG-3, VISTA, and TIM-3 components, which suppress T-cell activity and contribute to T-cell exhaustion. VISTA, expressed in approximately 60% of primary UM tumors, correlates with reduced CD8^+^ T-cell infiltration (Lamas et al., 2023). Additionally, UM exhibits epigenetic silencing of immune-stimulatory genes such as *CXCL9/10* through DNA modifications, while upregulating immunosuppressive factors, including IDO1, which catabolizes tryptophan—an essential amino acid for T-cell proliferation (de Vos et al., 2022).

Regarding metabolic alterations and oxygen-related signaling, hypoxia-related factors are upregulated in UM, promoting lactate production and creating an acidic microenvironment that impairs cytotoxic T-cell function (Yin et al., 2022). The cells modify their metabolic processes under stress conditions, predominantly utilizing oxidative phosphorylation (OXPHOS) while developing resistance to immune-mediated cell death mechanisms (Varney et al., 2025). Recent evidence suggests that this metabolic adaptation supports UM cell survival in nutrient-depleted conditions.

Chronic exposure to tumor antigens leads to progressive T-cell exhaustion, wherein sustained antigen stimulation gradually diminishes immune cell functionality. This form of adaptive resistance mechanism allows UM cells to escape immune detection despite initial immune responses, presenting challenges to maintaining treatment efficacy.

Chronic antigen exposure in UM leads to progressive immune cell depletion, primarily through enhanced activity of inhibitory markers such as PD-1 and CTLA-4, which significantly compromises their immunological function. Advanced genomic analyses reveal that specific immune cell populations become predominant while experiencing reduced diversity compared with treatment-responsive tumors. The immune system’s repertoire diminishes during extended tumor interactions (Durante et al., 2020; Tang et al., 2024). Additionally, tumor-derived extracellular vesicles containing signaling molecules such as PD-L1 and FAS ligand function as biological disruptors that induce premature death in activated immune cells, thereby diminishing the body’s inherent antitumor defenses (Dong et al., 2022).

While these biological adaptations demonstrate the tumor’s resilience, they also reveal potential therapeutic targets. Treatment strategies could include targeting alternative immune checkpoints such as LAG-3, modulating cellular energy metabolism, or combining conventional immunotherapies with epigenetic regulators. Notably, specific epigenetic modifiers demonstrate synergistic effects in initial studies (Synoradzki et al., 2024; Montazeri et al., 2023). Patient-stratified approaches utilizing biomarkers associated with DNA repair status or immune activation profiles may improve treatment selection accuracy, although clinical implementation requires additional validation across heterogeneous populations (Cao et al., 2021; Saint-Ghislain et al., 2022). Addressing this complex resistance mechanism requires comprehensive solutions that consider both the TME and systemic immune responses, necessitating interdisciplinary research collaboration.

## 3 Therapeutic modulation strategies in the TME of UM

The immunologically ‘cold’ TME in UM, characterized by low T-cell infiltration and dominant immunosuppressive cell populations, requires targeted modulation to enhance immunotherapy efficacy. Adjusting this environment to improve immune system recognition has become a key focus for overcoming treatment resistance. This section examines various strategies to influence cellular components, metabolic processes, and signaling interactions within the TME, drawing insights from recent experimental and clinical investigations.

### 3.1 Addressing immune-suppressing cell types

Certain immune cells and macrophage subtypes are notably abundant in UM cases, releasing substances such as IL-10 and TGF-β that hinder T-cell activity while promoting blood vessel formation (Kaler et al., 2022; Wang et al., 2020). Reducing these cells through specific inhibitors—such as those targeting cellular receptors—has demonstrated potential in laboratory models, improving T-cell presence and antitumor effects (Blomen et al., 2021). Similarly, modifying macrophage behavior using certain activating agents could enhance immune signaling and work synergistically with existing therapies targeting immune checkpoints (Goesmann et al., 2023).

These cells suppress immune responses through signaling pathways involving molecules such as CTLA-4 and adenosine. Combining antibody treatments targeting these molecules with adenosine pathway blockers has shown improved tumor control in animal studies, although further validation is needed (Heppt et al., 2019; Synoradzki et al., 2024).

### 3.2 Adjusting metabolic features

Tumors often exhibit reduced oxygen levels and acidic surroundings, which interfere with immune cell function. Inhibiting factors related to oxygen sensing or neutralizing acidity through basic compounds has been observed to restore T-cell activity in experimental settings (Yin et al., 2022; Varney et al., 2025).

An enzyme frequently overactive in these tumors depletes tryptophan—a substance critical for T-cell proliferation. Blocking this enzyme alongside immune checkpoint therapies has produced enhanced responses in early-stage trials, although outcomes remain variable (de Vos et al., 2022).

### 3.3 Enhancing T-cell infiltration and activation

UM tumors often lack chemokines (e.g., CXCL9/10) required for T-cell recruitment. Epigenetic drugs such as DNA methyltransferase inhibitors (e.g., decitabine) or histone deacetylase inhibitors (e.g., entinostat) reactivate CXCL9/10 expression, promoting T-cell trafficking (de Vos et al., 2022; Jespersen et al., 2019).

Chimeric antigen receptor (CAR)-T-cells targeting UM-associated antigens (e.g., PRAME, B7-H3) have demonstrated potent activity in preclinical models. HER2-targeted CAR-T-cells effectively eradicated ocular melanoma cell line and patient-derived xenografts in an IL-2 transgenic humanized mouse model (Forsberg et al., 2019). Co-administration with IL-2 or IL-15 cytokines enhances CAR-T persistence and tumor penetration in immune-excluded UM (Synoradzki et al., 2024).

### 3.4 Disrupting immune checkpoints and inhibitory signals

Beyond PD-1/CTLA-4, UM overexpresses alternative checkpoints such as LAG-3, VISTA, and TIGIT. Dual blockade of PD-1 and LAG-3 (e.g., relatlimab + nivolumab) has shown preliminary efficacy in metastatic UM, with an objective response rate of 15% reported in a phase II trial (Lutzky et al., 2021; Lamas et al., 2023). The study also points out that LAG-3 expression levels show a significant correlation with CD8^+^ T cell infiltration. Tumors with high LAG-3 expression exhibit richer immune cell infiltration, which may enhance the synergistic effects of dual blockade.

UM-derived EVs carry immunosuppressive cargo (e.g., PD-L1, FAS ligand) that induces T-cell apoptosis. Neutralizing EV release via Rab27a inhibition or blocking EV uptake with heparin sulfate mimetics reverses immunosuppression and enhances ICI responses (Dong et al., 2022).

### 3.5 Combining radiotherapy with immunotherapy

Radiation therapy induces immunogenic cell death and releases tumor-associated antigens, thereby stimulating systemic immunity. In UM, stereotactic radiosurgery (SRS) combined with anti–PD-1 therapy has demonstrated durable responses in liver metastases, potentially attributable to abscopal effects (Grynberg et al., 2022; Valaskova et al., 2024). Proton beam radiotherapy, which reduces collateral damage, enhances T-cell infiltration when combined with ICIs (Hager et al., 2019).

### 3.6 Epigenetic and transcriptional reprogramming

*BAP1* EZH2 overexpression suppresses tumor-suppressor genes and facilitates immune exclusion. EZH2 inhibitors (e.g., tazemetostat) counteract these effects, enhancing CD8^+^ T-cell infiltration and improving responses to anti–PD-1 therapy (Li et al., 2025).

Despite the promise of these strategies, significant challenges persist. The immunosuppressive complexity of UM requires combination therapies targeting multiple pathways concurrently. Moreover, patient stratification based on TME biomarkers (e.g., immune gene signatures, circulating cytokines) remains essential for identifying potential responders (Cao et al., 2021; Herrspiegel et al., 2023). Additionally, the optimization of dosing schedules and toxicity management, particularly in liver-dominant metastatic UM, warrants further investigation.

This multifaceted approach to TME reprogramming signifies a paradigm shift in UM treatment, advancing beyond single immune checkpoint blockade toward customized, mechanism-driven combinations that target the distinct immunosuppressive architecture of this malignancy.

## 4 Predictive biomarkers for immunotherapy response in UM

The identification of reliable markers for treatment efficacy in ocular malignancies remains an ongoing challenge. The complex and treatment-resistant nature of this condition complicates patient stratification, although contemporary approaches utilizing advanced diagnostic techniques and blood-based analyses have identified promising candidates warranting further investigation. This section synthesizes current knowledge regarding predictive markers, their clinical utility, and outstanding questions.

### 4.1 Genetic and molecular markers

*BAP1* gene alterations, occurring in approximately 50% of cases, are associated with accelerated disease progression and reduced immune activation. Tumors with these modifications demonstrate increased activity in specific immunosuppressive pathways, resulting in decreased immune cell infiltration and diminished responses to immunotherapy (Figueiredo et al., 2020; Kaler et al., 2022). In contrast, tumors without *BAP1* alterations may maintain partial immune competence and demonstrate improved survival rates during treatment (Durante et al., 2020).

Patients harboring *MBD4* gene mutations develop hypermutated tumors due to defective DNA repair mechanisms. These tumors exhibit increased immunogenic markers and enhanced therapeutic responses, with several studies documenting improved outcomes in advanced-stage patients receiving targeted therapies (Saint-Ghislain et al., 2022).

Gene expression profiles indicating immune system engagement, including those related to apoptotic pathways or immune cell populations, serve as valuable prognostic tools. One framework incorporating multiple apoptosis-related genes effectively stratified patients into distinct prognostic groups with significant survival differences (Cao et al., 2021). Additionally, another study identified an immune cell signature associated with improved outcomes in immunotherapy recipients (Sun et al., 2021).

Building on these findings, recent multi-omics studies have identified key subgroups and predictive markers in uveal melanoma. Co-loss of BAP1 with SF3B1 or EIF1AX mutations defines tumors with differing metastatic risks and unique cell-cycle and DNA-repair programs. In MBD4-mutant cases, promoter hypermethylation of antigen-presentation genes creates an “immune cold” despite high neoantigen burden. Finally, combined expression of exhausted CD8^+^ T-cell markers and myeloid-suppressive chemokines more accurately predicts response to checkpoint blockade than single-gene assays. These integrated biomarkers promise refined risk stratification and personalized therapeutic strategies.

### 4.2 TME features

High baseline CD8^+^ T-cell density and a low CD8+/Treg ratio correlate with ICI responsiveness. Single-cell RNA sequencing analyses demonstrate that clonally expanded, nonexhausted CD8^+^ T-cell populations are associated with improved survival (Durante et al., 2020; Tang et al., 2024). Conversely, elevated levels of myeloid-derived suppressor cells (MDSCs) or M2 macrophages indicate resistance (Wang et al., 2020).

Overexpression of alternative immune checkpoints, including LAG-3, VISTA, or TIGIT, in the TME indicates poor response to anti–PD-1 monotherapy. Combined targeting of PD-1 and LAG-3 (e.g., nivolumab + relatlimab) demonstrates increased response rates in LAG-3–high UM (Lutzky et al., 2021; Lamas et al., 2023).

### 4.3 Circulating biomarkers

Dynamic changes in ctDNA levels during treatment correlate with therapeutic response. Francis et al. demonstrated that ctDNA clearance after ICI initiation can predict radiographic response (AUC = 0.88) and prolong progression-free survival (HR = 0.32, p = 0.003) (Francis et al., 2024). However, this study is a single-case report that only tracked the treatment response of a 33-year-old male patient. Metastatic uveal melanoma itself exhibits high heterogeneity, and the results from a single case cannot be generalized to other patient populations. Observational data are susceptible to random fluctuations, and the lack of a control group in this study makes it impossible to determine whether the prolonged survival was attributable to immunotherapy itself or other confounding factors. Additionally, the absence of reported HR or other quantitative association metrics (e.g., odds ratio), as well as the lack of statistical analyses such as Kaplan-Meier curves or Cox regression, precludes assessment of the strength or significance of the association between ctDNA clearance and survival outcomes.

A six-protein serum signature (including IL-6, VEGF, and TIMP-1), identified by Herrspiegel et al. (2023), demonstrated predictive value for long-term survival in patients with metastatic UM (HR = 4.1, p < 0.001) (Herrspiegel et al., 2023). Elevated lactate dehydrogenase (LDH), an indicator of tumor burden and hypoxia, shows negative correlation with ICI efficacy (Liang et al., 2023).

### 4.4 Epigenetic and metabolic biomarkers

Hypermethylation of immunostimulatory genes (e.g., CXCL9, CXCL10) inhibits chemokine production, thereby limiting T-cell recruitment. Conversely, hypomethylation of PD-L1 or CTLA-4 loci indicates favorable responses to ICIs (de Vos et al., 2022).

High IDO1 expression in UM tumors or elevated serum kynurenine/tryptophan ratios indicate immunosuppression and ICI resistance. Research is ongoing to evaluate IDO1 inhibition in combination with anti–PD-1 therapy to counteract this mechanism.

### 4.5 Emerging multi-omics approaches

Integration of genomic, transcriptomic, and proteomic data has facilitated the development of composite biomarkers. Zhang et al. (2024) developed a prognostic model combining TRP channel-related lncRNAs (AC092535.4, LINC01637) with immune infiltration scores, demonstrating enhanced predictive accuracy (AUC = 0.92) compared with single-omics markers (Zhang et al., 2024). Similarly, Li et al. (2025) demonstrated that EZH2 overexpression correlates with immune exclusion and suggested EZH2 inhibition as a potential immunotherapy sensitizer (Li et al., 2025).

Heterogeneity and standardization: Biomarker studies frequently lack standardized protocols, resulting in inconsistent validation across cohorts. Dynamic monitoring: Static biomarkers cannot adequately track evolving resistance mechanisms. Serial liquid biopsies or imaging-based metrics (e.g., PET-CT) may provide solutions (Marko et al., 2020). Context-dependent utility: Biomarkers such as MBD4 status or HLA-A02:01 (for tebentafusp) maintain relevance only in specific therapeutic contexts (Saint-Ghislain et al., 2022; Damato et al., 2019).

Future research should emphasize on the prospective validation of multimodal biomarker panels and utilize artificial intelligence (AI) to integrate clinical, molecular, and imaging data. Collaborative initiatives, such as the UM Immunotherapy Biomarker Consortium, are essential for accelerating translation. Through refinement of biomarker-driven strategies, clinicians can optimize immunotherapy regimens, improve treatment sequencing, and enhance outcomes for patients with UM in the era of precision oncology.

## 5 Emerging combination therapies in UM

The modest efficacy of single-treatment approaches in UM has necessitated the exploration of combined strategies targeting multiple resistance mechanisms simultaneously. These methods integrate immune-modulating therapies with radiation treatment, precision medicines, epigenetic regulators, or novel immunotherapies to enhance antitumor immune responses, modify the TME, and address inherent treatment resistance. This section examines key combination approaches under investigation.

### 5.1 ICIs with targeted therapies

Tebentafusp, the first approved treatment for advanced UM, as a gp100 × CD3-targeting bispecific ImmTAC, it directs T-cells to target cancer cells expressing specific markers (Sacco et al., 2024). The 3-year efficacy and safety results from a (Alam et al., 2025; Agrawal et al., 2023) open-label phase 3 trial demonstrated sustained overall survival benefit with tebentafusp in HLA-A*02:01-positive adults with previously untreated metastatic uveal melanoma, showing a 27% 3-year survival rate compared with 18% in the control group (Hassel et al., 2023). Combined administration with ICIs such as nivolumab in genetically compatible patients has demonstrated improved survival outcomes—specifically, a 1-year survival rate of 73% compared with 58% with monotherapy (Damato et al., 2019; Dimitriou et al., 2025). This enhancement results from tebentafusp’s T-cell activation properties complementing checkpoint inhibitors’ reduction of cellular exhaustion. However, the observed therapeutic benefits of the combinatorial regimen should be interpreted with caution due to the small cohort size, which might lead to overestimation of treatment effects.

Considering UM’s dependence on specific signaling pathways, the combination of enzyme-blocking agents with PD-1 inhibitors demonstrates potential for enhancing immune cell infiltration while reducing immunosuppressive factors. Initial trials documented response rates approaching 20% for these combinations, although the management of treatment-related adverse effects requires optimization (Khan and Carvajal, 2020).

### 5.2 Immune therapies with epigenetic adjusters

Epigenetic modifications that contribute to immune-evasive tumor environments can be targeted using chromatin-modifying drugs. The combination of histone deacetylase inhibitors with PD-1 blockers has shown the potential to restore immune signaling pathways in clinical trials, achieving response rates of approximately 15% (Jespersen et al., 2019). Additionally, DNA methylation inhibitors can enhance antigen presentation, increasing the efficacy of CTLA-4 inhibitors (de Vos et al., 2022).

In melanoma, hyperactive chromatin regulators facilitate immune exclusion. Preclinical studies have shown that combining EZH2-targeting agents with PD-1 inhibitors increases infiltration of immune cells into the tumor and extends survival, with clinical trials currently evaluating these effects (Li et al., 2025).

### 5.3 Immune therapies with metabolic interference

Metabolic enzymes that shape immunosuppressive environments can be inhibited to enhance treatment responses. Initial studies examining IDO1 inhibitors in combination with checkpoint blockers have demonstrated modest improvements in clinical outcomes, although the underlying mechanisms require further investigation. Understanding the interaction between metabolic reprogramming and immune activation remains essential for optimizing such combinations.

UM evades immune surveillance by upregulating the IDO1 enzyme. The phase 1/2 ECHO-202/KEYNOTE-037 study demonstrated that the combination of IDO1 inhibitor epacadostat with pembrolizumab was well-tolerated and exhibited antitumor activity. However, this phase 3 study (NCT02752074) yielded negative results—in 706 patients with advanced melanoma, the combination therapy failed to improve progression-free survival or overall survival compared to pembrolizumab monotherapy (Mitchell et al., 2018; Long et al., 2019).

Regarding arginase-related approaches, Arginase-1 produced by specific immune cells decreases arginine availability, creating suboptimal conditions for T-cell function. Animal studies combining the arginase-blocking compound CB-1158 with nivolumab demonstrated dual effects: immune cell reactivation and reduced metastatic growth in liver tissues (Blomen et al., 2021).

In radiation combination strategies, stereotactic radiation applied to liver lesions in conjunction with anti–PD-1 therapy yielded response rates of approximately 25% and median survival periods approaching 18 months (Grynberg et al., 2022; Valaskova et al., 2024). This approach leverages radiation-induced tumor antigen release while immunotherapy enhances systemic responses. Additionally, hepatic perfusion techniques delivering concentrated chemotherapy to the liver demonstrated disease control rates of 35% when combined with ICIs, although the underlying mechanisms warrant further investigation (Arulananda et al., 2019).

Novel cellular therapies incorporate modified immune cells targeting tumor markers such as PRAME or B7-H3. These engineered cells, when administered with PD-1 inhibitors, sustained antitumor activity longer in preclinical models (Synoradzki et al., 2024). Similarly, T-cell receptor therapies targeting melanoma-specific proteins achieved response rates of 30% in early trials among patients with compatible genetic markers (Strobel et al., 2022).

Triple-therapy approaches combining immune agents, targeted drugs, and epigenetic modifiers achieved disease control rates of 50% through multiple synergistic mechanisms—redirecting immune cells, blocking inhibitory signals, and modifying TME conditions (Montazeri et al., 2023). More sophisticated regimens sequentially applying radiation, metabolic pathway inhibitors, cellular therapies, and checkpoint blockers demonstrated potential in eliminating liver metastases by addressing both energy utilization patterns and immune-evasion strategies (Varney et al., 2025).

Current challenges include managing intensified side effects from combination therapies, necessitating careful dosage adjustments (Khimani et al., 2022). The identification of predictive biomarkers—including genetic mutation status and immune compatibility markers—remains essential for patient selection (Saint-Ghislain et al., 2022; Cao et al., 2021). Furthermore, determining optimal treatment sequences across modalities requires additional research to maximize clinical benefits while minimizing risks.

## 6 Future directions and innovative strategies in UM

Expanding knowledge of UM biology and treatment resistance has prompted the investigation of novel strategies to improve outcomes. This section outlines emerging technologies and therapeutic approaches that may transform UM management, although many require further validation.

### 6.1 Precision medicine and patient stratification

The integration of multiple biological data types supports the development of composite biomarkers for personalized care. Zhang et al. (2024) demonstrated that combining genetic markers with immune system scores can predict treatment responses with high accuracy (Zhang et al., 2024). Additionally, AI tools analyzing medical images and blood samples may enhance risk categorization, although practical applications remain uncertain (Wan et al., 2023; Herrspiegel et al., 2023).

Although UM typically exhibits limited genetic variability, rare cases harboring specific mutations may respond to tailored vaccine strategies or modified immune cells (Saint-Ghislain et al., 2022; Strobel et al., 2022). Advances in cellular analysis techniques may help identify unique markers in advanced-stage UM, paving the way for customized cell-based treatments (Durante et al., 2020).

### 6.2 Next-generation immunotherapies

#### 6.2.1 Dual-target antibody therapies and engineered immune cells

Treatment successes have increased interest in antibodies targeting UM-specific markers. Engineered immune cells capable of recognizing multiple targets have demonstrated improved tumor eradication in early studies, although their clinical relevance remains to be validated (Synoradzki et al., 2024).

NK cells from donors modified with specialized receptors may bypass compatibility issues while attacking tumors. Preclinical data indicate these cells both destroy UM cells and release substances that alter the tumor environment, although long-term effects remain uncertain (Kong et al., 2022).

### 6.3 Epigenetic and metabolic adjustments

EZH2 (e.g., tazemetostat) and BET (e.g., JQ1) inhibitors reverse immune exclusion by reactivating silenced tumor-suppressor genes (e.g., HLA class I) and chemokine production (Li et al., 2025; de Vos et al., 2022). Combining these agents with ICIs is under clinical evaluation.

UM’s dependence on oxidative phosphorylation (OXPHOS) renders it susceptible to OXPHOS inhibitors (e.g., IACS-010759). Preclinical studies demonstrate that OXPHOS inhibition works synergistically with anti–PD-1 therapy by triggering immunogenic cell death and decreasing lactate-driven immunosuppression (Varney et al., 2025).

### 6.4 Technology-driven therapeutic optimization

Advanced computational tools: Machine learning systems trained on medical imaging data and biological datasets can evaluate the risks of disease progression and predict treatment responses. A 2023 project developed pattern recognition models that identified novel prognostic markers with significant reliability, achieving outcomes comparable to expert assessments (Wan et al., 2023).

The analysis of spatial distributions of different cell types within affected tissues reveals localized resistance mechanisms. This approach could guide precisely targeted interventions, such as direct injections of therapeutic agents or specialized viral treatments, as investigated in recent experimental work (Tang et al., 2024).

### 6.5 Innovative clinical trial designs

Flexible clinical trial designs that allow simultaneous evaluation of multiple therapies using real-time biological data may accelerate treatment development, particularly for rare disease subtypes. Recent initiatives have demonstrated the viability of applying this model to rare conditions characterized by specific genetic markers (Dimitriou et al., 2025).

Multicondition therapeutic studies: Liver involvement in UM exhibits biological similarities to other hepatic diseases, particularly regarding blood vessel formation processes. Studies focused on liver-targeted combination therapies might identify beneficial interactions across different diseases, as indicated by earlier exploratory trials (Blomen et al., 2021).

### 6.6 Prevention of metastasis and early intervention

Monitoring blood-based biomarkers after primary interventions enables the detection of residual disease signals, allowing early administration of adjuvant therapies such as immune-modulating drugs or precision medications. Recent reports demonstrate the potential of this approach in enhancing long-term outcomes (Francis et al., 2024).

Patients with specific genetic risk factors might benefit from preventive liver-targeted therapies designed to eliminate microscopic disease spread before it becomes detectable via imaging, as examined in recent clinical reviews (Grynberg et al., 2022).

Intensive combination regimens require comprehensive support protocols and dosage adjustments guided by biological indicators, necessitating close monitoring of patient responses (Khimani et al., 2022). International research networks must standardize biomarker measurement techniques and integrate datasets to address challenges posed by the condition’s rarity. Streamlined approval processes could expedite access to promising therapies for underserved patient populations, while maintaining thorough evaluation standards.

## 7 Conclusion

Uveal Melanoma (UM) remains a challenging cancer, characterized by distinct genetic features, immune resistance, and limited response to standard treatments. While immunotherapy has significantly improved outcomes in other cancers, its effectiveness in UM is low, with fewer than 10% of patients showing positive responses. This resistance is driven by factors such as limited genetic changes, immune evasion mechanisms, and a tumor microenvironment that suppresses immune activity. However, recent advances in understanding UM biology have opened new possibilities for treatment development.

The emergence of targeted therapies that direct immune cells to attack cancer-specific markers has shown promise in improving survival, particularly in patients with certain genetic traits. Combining immunotherapy with other approaches, such as drugs that modify cell behavior or radiation therapy, has demonstrated early potential by altering the tumor environment and reducing immune suppression. Efforts to identify biomarkers are helping to personalize treatment strategies.

Looking ahead, integrating advanced genetic profiling, medical imaging analysis, and tissue studies could transform UM patient care. Machine learning models analyzing patient data may improve predictions of disease progression and treatment responsiveness. Additionally, new therapies, such as modified immune cell treatments and dual-target antibody approaches, aim to address genetic limitations and improve tumor targeting. While challenges remain, including treatment toxicity and the rarity of UM, international collaboration and advancements in clinical trial design will be crucial for accelerating progress.

In conclusion, the future of UM treatment depends on combining scientific innovation, technological advancements, and global collaboration. By targeting the immune-resistant tumor microenvironment, using predictive biomarkers, and developing novel immune-based therapies, the management of UM is shifting from uncertainty to cautious optimism, offering potential improvements in both survival and quality of life for patients worldwide.
